# Yoga in adult cancer: an exploratory, qualitative analysis of the patient experience

**DOI:** 10.1186/s12906-015-0738-9

**Published:** 2015-07-22

**Authors:** Marcy McCall, Sally Thorne, Alison Ward, Carl Heneghan

**Affiliations:** Kellogg College, University of Oxford, 62 Banbury Road, Oxford, OX2 6PN United Kingdom; School of Nursing, University of British Columbia, T213-2211 Wesbrook Mall, Vancouver, V6T 2B5 British Columbia; Department of Primary Health Care Sciences, University of Oxford, New Radcliffe House, 2nd floor, Walton Street, Jericho, OX2 6NW United Kingdom

**Keywords:** Yoga, Cancer, Patient, Qualitative analysis, Interview, Interpretive description

## Abstract

**Background:**

Some patients receiving treatment in conventional health care systems access therapeutic yoga outside their mainstream care to improve cancer symptoms. Given the current knowledge gap around patient preferences and documented experiences of yoga in adult cancer, this study aimed to describe patient-reported benefits, barriers and characteristics of programming for yoga practice during conventional treatment.

**Methods:**

In depth semi-structured interviews (*n* = 10) were conducted in men and women recruited from cancer care clinics in Vancouver, Canada using a purposive sampling technique. The exploratory interviews were audio-recorded, transcribed and analyzed using Interpretive Description methodology and constant comparative analysis methods.

**Results:**

Four themes emerged from the data to address our research objectives: patient-perceived benefits of yoga, reasons and motivations for practising yoga, hurdles and barriers to practising yoga, and advice for effective yoga program delivery in adult cancer. Several patients reported yoga reduced stress and other symptoms associated with cancer treatment. Thematic analysis found the social dimension of group yoga was important, as well as yoga’s ability to encourage personal empowerment and awareness of physical body and self. Barriers to yoga adherence from the patient perspective included lack of time, scheduling conflicts and worries about financial burden.

**Conclusion:**

This small, diverse sample of patients reported positive experiences and no adverse effects following yoga practice for management of cancer and its symptoms. Results of this qualitative study identified patient-reported preferences, barriers and characteristics of yoga intervention optimal during adult cancer treatment.

## Background

Cancer patients in conventional treatment usually receive one or more of radiotherapy, chemotherapy, surgical intervention and hormone therapy [1]. In addition to this, nearly half of all patients supplement this care with complementary therapeutic interventions including vitamin supplementation, herbal teas and medicines, nutritional advice, acupuncture, prayer and meditation and yoga [2, 3]. Yoga is one complementary therapy used by cancer patients because it helps to lift mood and enhance wellbeing [4, 5].

As a traditional discipline derived from Indian culture, yoga includes aspects of physical postures (*asana*), breathing techniques (*pranayama*), and meditation (*dhyana*), chants (*mantras*) and wisdom teachings (*sutras*) to encourage health and relaxation [6, 7]. Yoga exists in the context of westernized health care as a complementary or physical therapy and exercise movement technique including aspects of religious philosophy [8]. A recent overview of systematic reviews identified Hatha yoga as effective for decreasing anxiety, depression and pain in acute and chronic health conditions of adult populations with no adverse effects [9]. According to this overview, yoga intervention improves psychological health outcomes (anxiety, depression, distress, stress) and may improve quality of life for cancer patients. Additional systematic reviews in adult cancer suggest it has positive effects in women with breast cancer to reduce fatigue, nausea and quality of life [10–12].

As the frequency of research conducted in yoga intervention for health care increases [13] and quantitative findings begin to generate data to support its use in adult cancer [14], the contextual factors of yoga delivery are increasingly important but not well understood [15]. The patient-reported experience and their description of how, when and why yoga practice could be helpful to patients in a clinical setting has not been documented in a scientific way.

The primary objectives of this study were to gain knowledge of patient experiences of yoga including their reported risks, barriers and other social, environmental or personal factors that influence initial participation and adherence to regular yoga practice. The secondary objective was to understand the potential role and specific programming characteristics of yoga delivery for adult cancer in a conventional setting.

## Methods

The semi-structured interviews were performed under naturalistic conditions in adult men and women (18 years of age or older), receiving complementary care alongside physician-supervised treatment for a cancer diagnosis. In-person and telephone interviews were conducted over a six-month period from September 2013 to March 2014. English-speaking patients receiving conventional treatment in Vancouver, Canada were recruited for this study through poster advertisements in two cancer hospitals and three complementary care clinics. Patients self-reporting non-debilitating co-morbidities alongside cancer were included.

### Sampling and sample size

A purposive sampling technique was implemented to capture a spectrum of data [16]. Participants were selected based upon knowledge about their age, gender, characteristics of disease and therapeutic use of yoga and other complementary therapies during cancer treatment. The recruitment end-point was defined as the moment when interviewer (MM) could identify conceptual themes or patterns with sufficient data. The sample size parameters were set to meet resource limitations and quality standards and included minimum 10 hours of interview time collected from a patient sample size minimum (*n* = 8) and maximum (*n* = 20).

### Ethics

Ethical approval was provided by the University of British Columbia Behavioural Research Ethics Board and University of Oxford Tropical Research Ethics Committee (Reference: 517–13). An electronic letter of informed consent was sent to participants more than 48 hours before the interview meeting. Signed consent was obtained at the time of interview in paper form (*n* = 8) or audio recorded (*n* = 2).

### Data collection and analysis methodology

The study design and qualitative analysis were informed under interpretive description (ID) methodology [17, 18]. ID methodology was selected for its emphasis on pattern recognition and its aim to provide descriptive interpretation across cases for complex experiential health care questions [19]. Theme and pattern recognition procedures were followed using the Qualitative Analysis Guide of Leuven (QUAGOL) (MM) [20]. The thematic analysis used an iterative process of constant comparison methods [21] to identify emergent patterns (MM, ST). The comprehension of data meanings assisted to theorize relationships and led to the recontexualization of data into findings (MM, ST) [22]. Coding data for theme recognition was performed using NVivo 10TM (QSR International, Doncaster, Victoria, Australia) software (MM).

A reflexive journal [23] of interviewer’s field notes and observations was used to document the reflexive process. The field notes included interviewer’s opinions and perceptions of what was being shared by participants, and the observations included non-verbal communication or environmental cues. The reflexive journal required the interviewer (MM) to consider the potential interpreter bias and the ‘role’ of the researcher in the data collection process. Interviewer’s recall bias, transcription or other human processing errors were minimized through immediate audio-recorded journaling and verbatim transcription. The data were analyzed progressively over time (6 months) under supervision (CH, AW, ST) to avoid misclassification or misrepresentation.

The interviewer script was modified over time to include more refined questions (see Table 1 for the original set of questions, and Table 2 for the final interview script). Anonymous audio files were recorded on a digital memory card and immediately deleted upon transcription to word documents (.docx). Digital files remain encrypted and stored under password protection (AW, MM).

**Table 1:** Original version of interview script

1. Tell me about your cancer diagnosis and treatment.
2. Why did you decide to seek complementary care for your cancer treatment? Are there other reasons?
3. I am curious about your thoughts on yoga. How do you feel about yoga?
4. Can you describe an experience in yoga, either before or now, during your cancer?
5. How often would you say you practice yoga now that you have cancer?
6. Why have you (or have not) practised yoga during your cancer treatment?
7. Tell me about your fears or concerns about doing yoga.
8. What kinds of things stop would have stopped you from practising yoga this week?
9. If you could imagine the perfect time and place for yoga, what would it be? What about before or after your cancer treatment? At home? In a clinic?

**Table 2:** Final version of interview script

1. Tell me about your cancer diagnosis and treatment.
2. Why did you decide to seek complementary care for your cancer treatment? Are there other reasons?
3. What complementary care therapies have you tried? What are you currently doing?
4. Have you experienced any harm or benefits from complementary therapy?
5. I am curious about your thoughts on yoga. How do you feel about yoga?
6. Why have you (or have not) practised yoga during your cancer treatment? Tell me about your fears or concerns about doing yoga.
7. What kinds of things stop you from practising yoga?
8. If your doctor recommended yoga, would you be more inclined to do it?
9. Who do you think should practise yoga and when should they do it?
10. Do you think yoga would be helpful if practised before or during a cancer treatment?
11. What kind of person might not do yoga?
*Yoga-users only*:
12. What are the most important parts or components of yoga for you?
13. What is it about the atmosphere or class structure of yoga that works for you?
14. Has doing yoga ever aggravated a symptom or health-related issue for you?
*All interviewees:*
15. Can you tell me about the emotional or spiritual impact of cancer on your life?
16. How has cancer or its treatment impacted you financially?
17. Is there anything you would like to change about your treatment plan?
18. Is there more you would like to share about your experience with cancer and its treatment?

## Results

Ten in-depth interviews were conducted in adult cancer patients receiving complementary care alongside conventional treatment in Vancouver, Canada. Individual interviews took place in an integrated cancer care clinic (*n* = 7), over the telephone (*n* = 2) and patient’s home (*n* = 1). The interviews lasted between 43.4 and 103.5 minutes with an average of 75.1 minutes for women and 49.4 minutes for men interviewees. A total of 11 hours 15 minutes of interviews data were analyzed for themes and patterns.

### Participant demographics, cancer and treatment characteristics

Men (*n* = 3) and women (*n* = 7) cancer patients receiving conventional and complementary care for a current diagnosis participated in the study. Age of participants ranged from 44 to 71 years (average age of 60.1 years). Participants were retired (*n* = 5), on disability leave (*n* = 3), working at reduced hours (*n* = 1) or employed full-time (*n* = 1). The highest attained level of education for one participant was a secondary school diploma, the other nine participants completed college or university degrees. Study participants were receiving treatment for cancerous tumours occurring in the breast (*n* = 5), prostate (*n* = 2), bone marrow (*n* = 2), and skin (*n* = 1); at diagnostic stages I (*n* = 1), II (*n* = 2), III (*n* = 1), IV (*n* = 3) and unreported (*n* = 3). Seven of the 10 informants were participating in a modified yoga practice during cancer treatment. Four of these seven patients had never practised yoga prior to receiving a cancer diagnosis. Of the patients using yoga as a complementary therapy, six men (*n* = 2) and women (*n* = 4) attended the same 60-minute restorative yoga class provided three times per week in a private cancer care facility. Participants reported use of other complementary therapies including: exercise, massage, acupuncture, vitamin supplementation (i.e. vitamin C, vitamin D), talk therapy, nutritional counselling and medicinal herbs (i.e. mistletoe, marijuana).

### Thematic analysis

Four themes emerged from the data to address our research objectives: patient-perceived benefits of yoga, reasons and motivations for practising yoga, hurdles and barriers to practising yoga, and advice for effective yoga program and delivery for adult cancer. See Table 3 for a summary of subject areas and identified patterns. This thematic summary of personal accounts from 10 cancer patients points to their perception of yoga and their ideas about possible considerations for inclusion of yoga opportunities within cancer settings.

**Table 3:** Thematic analysis of interview data

**Theme**	**Category**	**Pattern**	**Supporting Statement**
Patient-perceived benefits of yoga	Psychological and emotional wellbeing	Reduces my stress	"It's that calming down, and yoga is helpful for that…stress reduction is a big one…(after yoga) I feel more relaxed but energized at the same time. It's a really, really nice feeling….Yoga is terrific!"
		Feelings of peace and quiet	"My yoga practice has been huge, because it has allowed me, even in this kind of turmoil, to find peace."
		Increases acceptance of self and world around me	"I find it very comfortable and rewarding…I just felt much calmer and more accepting."
	Physical wellbeing and body awareness	Improves strength flexibility	"I feel stronger in my body, if nothing else. I don’t know if it's dealt with the fatigue so much, but it's certainly made me feel more solid."
		It's like exercise, but better	"I think because it's so adaptive and gentle, it's a really beneficial way to get our bodies moving again and the environment of a yoga class is a safe place."
	Facilitates groups support and social cohesion	Comforting to be with people who know about cancer	" Although my friends are supportive…the people here understand more of that, more of what you are going through. And it's nice to compare notes."
		A way to socialize, and meet interesting people	"The community aspect…we socialize and hike and it's nice to hear what the other people are doing…I think that's big factor…”
Reasons and motivation for practising yoga	Seeking a non-medical approach	Desire for a holistic, integrated way of thinking	"I want to pursue a more holistic approach to anything in my life."
		Self-identify as a healthy person, not a patient	"That was my first surgery since I had my tonsils out when I was ten. I don’t want to just be a medical patient."
	Maximizing treatment plan	To do all I can to prolong and enjoy my life	"I think it has been an important part of my treatment. And just the feeling that you are doing everything that you can to prolong your life. And your quality of life, too."
		Creating a treatment plan that suits me	"My qualms with the conventional treatment model is right now it's off the shelf, 'oh, you've got prostate cancer? Here, do this or that', it's not customized and personalized…that's why I've chosen my own personalized treatment of different practices."
	Personal responsibility for illness and wellness		"Surgery, radiation and chemo are passive forms of healing, you turn yourself over to somebody to be healed, and these other forms are active healing…I'm actively involved in my own health and healing, not waiting for some saviour on the outside to make me better."
Hurdles and barriers to practising yoga	Logistics	Lack of time	"You have to pick Suzie up at day care and Johnny needs to go skating and somebody's got to make dinner, where do you find time?"
		Cost and accessibility of yoga	"Time, cost and location. Because this isn't a great location, parking is difficult."
		Competing priorities during acute treatment	"Looking back on it, it would have been good for me to go to those yoga classes (during chemotherapy)…but I didn't until the end of chemo…there was so much going on."
	Personal factors	Lack of discipline	"Discipline. I mean these five Tibetan rights that I could easily do every morning…but I got on it for a month or so and then slid away from that."
Recommendations for effective yoga program and delivery	Instruction	Expert instruction with a gentle and predictable class sequence	"We all get in the room and we usually throw our legs up the wall…and then she (the instructor) comes in…and she brings us back down and off we go…it's a pretty good routine…and it's yoga…a good stretch, a good workout."
	Supporting a home-practice	Training needed about how to do brief sessions	"It doesn't have to be long, but it has to be every day."
	Online or video-led yoga	Not desirable, would miss the instructor-led component	"You need to have somebody instructing I think. Otherwise it's very easy not to turn it on…You go to the class and you know for the hour…you are going to be concentrating on doing the yoga."
	Cancer-specific yoga	Cancer-only groups make me feel more comfortable	"That this is like our private little club of people who have cancer and you don’t have cancer, you don’t belong here. I’m exaggerating that. I don’t feel that strongly but it is sort of like what are you doing here really?"
		Negative experience with community-based classes	"There's other yoga places I've gone to and they say, 'go, go, go, go!' You know what I'm saying? [laughter] And I don't wanna be ah 'go, go, go' because, you know, I probably will stretch my muscles too fast. I need to be gentle with myself right now and I feel that that's a place where I can do it."

Patient-perceived benefits included psychological and emotional wellbeing, physical wellbeing and body awareness, social cohesion and additional group support from peers. Yoga-users and patients using complementary therapy both reported a sense of personal responsibility for their health and outcomes, and were seeking a non-medical approach and a way to maximize their individual treatment. Practical struggles such as lack of time, cost, lack of personal discipline or accessibility are perceived barriers to adherence to yoga. It was suggested the effective delivery of yoga in a clinical setting includes expert in-person instruction of gentle postures, breathing techniques and meditation in a cancer-specific group. Several patients identified the ambience created by instructors and the yoga space as an important factor in their enjoyment and adherence to a yoga program.

#### Perceived benefits of yoga

The patients using yoga as a complementary therapy in adult cancer (*n* = 7) reported positive benefits in terms of physical, psychological and social wellbeing. The most commonly reported direct benefit of yoga was its breath-related awareness of mind and body. Patients said yoga taught them about body awareness and encouraged them to accept ‘what is,’ giving them a sense of connection and calmness in their mind and body. One woman who tried yoga for the first time following her cancer diagnosis shared:

I found the meditation quite relaxing…the breathing for relaxation really helped, too. I think you are more aware of your breath and you are more aware of all of your muscles and just the way that your body moves. I think you are just more aware…I just feel really satisfied. It makes me feel good physically and emotionally and mentally.

Another woman explained yoga was “very comfortable and rewarding − I just feel much calmer and accepting” after a class. Other patient-reported benefits include: improved stress management, better sleeping habits and a new capacity to experience peacefulness.

Some respondents described yoga’s usefulness to improve physical strength, and decrease stiffness and body aches associated with cancer and its treatment. One woman felt: “when my body was aching, I would do yoga and it would help,” and another woman said: “I certainly feel stronger in my body…more solid.”

Participants also talked about yoga providing social benefit. Men and women reported that group dynamics were an “important” aspect of yoga, and felt especially comforted by participating in cancer-specific yoga classes. All patients practicing yoga during their cancer treatment (*n* = 7) felt they received a great deal of social support from their yoga community. One man reported:

…besides the benefit of the exercise there’s a social benefit as well because some of these people… like we hike together and we have lunch together and things like that, so it’s like a little community…this I consider to be as much a family as anything.

The social benefits of participating in yoga appeared to be reinforced by the shared experience of having cancer. Patients said doing yoga in a cancer-specific group was especially helpful to learn about what their peers have found helpful and sharing their feelings about cancer and its treatment.

#### Patient-reported reasons and motivation for practising yoga

The prevailing attitude of respondents was their determination to do their best and everything they could to improve their chance of survival. The health locus of personal control of interviewees was high, and most perceived cancer as a teacher in their life to do things better. One man with prostate cancer made the distinction between passive and active healing, where:

…surgery, radiation and chemo, are passive forms of healing, you turn yourself over to somebody to be healed, and these other forms are active healing…I’m actively involved in my own health and healing, not waiting for some saviour on the outside to make me better.

These users of complementary care and yoga were seeking a non-medical approach, including holistic, integrated ways of thinking with expectations for a personalized treatment plan. A woman with breast cancer felt that doing yoga was her way of committing to life as a person, not a patient:

I want to pursue a more holistic approach to anything in my life. I’m someone who’s not ever sick…that was my first surgery since I had my tonsils out when I was ten. I don’t want to just be a medical patient.

A first-time yoga practitioner following her breast cancer diagnosis chose yoga for its comforting, gentle approach to physical movement:

…it’s so adaptive and gentle, it’s a really beneficial way to get our bodies moving again and the environment of a yoga class is a safe place. You get some personal instruction from someone who can help you if you’re uncertain and you get lots of choices about you can do it this way or this way and this might work better for you.

The customization of yoga was appealing to participants. Reporting yoga participants enjoyed the non-competitive atmosphere and felt motivated by the reassurance received from expert instructors to follow their own rhythm and listen to their bodies.

#### Hurdles and barriers to practising yoga

Scheduling, transportation, lack of time and competing priorities were listed as key barriers to attending regular yoga class. One man would have liked to participate in yoga at an integrated health centre but said “the parking is always a bear…and I do find it stressful…to get in my fitness classes or doctors appointments or whatever. So while the yoga can be a de-stressor, the drive home can just sort of ruin all that!” Personal factors and financial concerns were also identified as potential limitations. A woman with two children admitted:

The only trouble is, honestly the time thing. I mean…the financial, too…because the extra stuff is expensive…if you have to pay the mortgage, work 9 to 5 and you have to pick Suzie up at day care and Johnny needs to go to skating and somebody’s got to make dinner, where do you find time?

Participants believed that online yoga or video-led yoga would not be an attractive alternative to address logistical and scheduling burdens. A woman who has sampled video sessions for yoga found them uninspiring, and difficult to follow due to the lack of in-person instruction. Furthermore, participants (*n* = 4) reported being more apt to come to class when they made plans with peers to socialize before or after the session. Online or video yoga may not be ideal for motivation or adherence because of the missing social component.

According to several respondents, the yogic environment in their integrated oncology centre included a safe, quiet space with expert instructors who appreciated the concerns of cancer patients. A concern over personal treatment and stigma in public yoga classes was identified when asked about attendance in community-based classes. One cancer patient had this to say about participating in public classes:

I will go bald at yoga - because I'm not going to be able to wear a hat or a scarf and I don't like the wigs. I know that would make me and other people uncomfortable in a yoga class, say at my community centre.

#### Advice for effective yoga program and delivery

A description of the ideal yoga program from patients included a focus on breathing techniques, a long warm-up with a variety of postures (asanas) and inclusion of savasana (supine relaxation) at the end of class. Many informants also noted that meditation is a challenge at first, but that it is a useful, transferable skill during treatment sessions and in daily life. When asked about their favourite components of yoga, participants were not able to distinguish between the most important aspects of yoga practice. Several respondents (*n* = 6) felt the various components of yoga, including the combination of postures, breathing and visualization techniques during meditation are all important aspects of a yoga class. When asked about their favourite part of yoga, one male participant said: “I don’t know, it depends. I guess it all kind of works…it’s yoga.”

Several patients (*n* = 6) said they would appreciate recommendations from oncologists or health care practitioners about specific locations or types of yoga that would be suitable for their condition. Some respondents also talked about the likability of the yoga teacher, and how it was a fundamental concern to the successful delivery of a yoga class. One woman said:

I think it's like anything: if you resonate with the teacher you are going to do much better. It's like any subject you ever take. If the teacher and you kind of work you are going to do well in the course.

Restorative or yin types of yoga practices appealed to the greatest number of participants for program suitability at all stages of cancer treatment and recovery. Restorative and yin yoga classes are slow-paced, sometimes referred to as ‘active relaxation’ where by with props, the body is prompted to relax and assist people in the recovery of illness or injury [24, 25]. Other participants enjoyed the physical challenge of some yoga postures and added that a yoga class was unique in that it allowed them to do more or less activity, depending on how they felt on a particular day.

A flexible and easily accessed yoga class was believed as critical to patient adherence to yoga class; respondents in general felt their schedules were difficult to accommodate, especially during early treatment with numerous medical visits, work and family commitments. A small user fee for yoga services delivered in a clinical setting would be acceptable to all patients and is currently implemented in the integrative health care centre where the majority of respondents attended yoga class. One woman felt the costs associated with practicing yoga in a private cancer clinic were reasonable, and much less than the market rate: “…fees are very low here…I think they do that intentionally…because the cancer patients aren’t working and lot of us don’t have sources of income.” Another woman who attended yoga in a conventional care centre also said: “…it does cost money, but it is subsidized…I don’t know who pays for it actually, but I do think it has been an important part of my treatment.” However, the interview reported the yoga program delivered in the conventional hospital was eventually suspended, as administrators said the availability of space became an issue.

Overall, participants agreed yoga delivery in a conventional cancer facility would be a practical solution and could help to improve the cancer treatment experience. The respondents said a yoga class of 60 minutes was typical, although some participants felt an extension of 15 to 30 minutes was desirable. One woman suggested, “if the space was quiet it might be nice to have a positive experience in a building that is usually quite stressful,” and others felt yoga delivered within the same building as their cancer treatment might reduce concerns about accessibility. Some respondents thought short, meditative or pranayama-focused yoga sessions (20 to 40-minute sessions) in the waiting room before or during radiation or chemotherapy treatment could help to reduce their psychological stress.

## Discussion

This set of exploratory interviews identified four thematic areas of interest for yoga in adult cancer: patient-perceived benefits of yoga, reasons and motivations for practising yoga, hurdles and barriers to practising yoga, and advice for effective yoga delivery in adult cancer. Several men and women reported yoga reduced stress, fatigue and other negative symptoms associated with cancer treatment. As well, yoga was reported by patients as a means to encourage personal empowerment and improved awareness of physical body and self.

Adverse events or negative physical or emotional side effects of yoga were not reported in this sample. Barriers to yoga adherence from the patient perspective included lack of time, scheduling conflicts and worries about financial burden. Community-based yoga or yoga delivered online or video-based were not frequently accessed by this group of cancer patients, due to a perceived stigma by class members who do not have cancer, and worries about the non-specialized instruction they might receive.

The social dimension of group yoga was important to the interviewed patients, and this aspect of yoga is a potential therapeutic tool in cancer care. In previous literature, “a decrease in the level of distress through social support is also expected to lead to the improvement of immune competence”[26]. The extent to which yoga might encourage social wellbeing and improve morbidity or mortality of cancer patients is an area for future scientific investigation.

The class characteristics of an ideal yoga intervention in a conventional care centre included: flexible scheduling of short, pranayama-based exercises to relieve stress (20 to 30 minutes) to regular three 60 to 90-minutes sessions per week of restorative yoga; that included some aspect of breathing exercises, meditation techniques and physical postures.

### Strengths and limitations

The ID methodology guided this preliminary exploration of yoga in adult cancer in the context of health and complementary care. The interview methods allowed the researchers to form a coherent perspective based upon an inductive reasoning approach. The qualitative data gathered across 10 interviews with adult patients of cancer is the first known systematic documentation of how yoga intervention is perceived by a group of patients seeking complementary care. Although the findings are important, there are some serious study limitations to be considered.

First, the sample size was limited by slow recruitment, a short recruitment period (6 months), and practical limitations including time, financial and human resources constraints. The extremely low response rate could be attributed to lack of time, priority or interest on the part of patients, or, lack of willingness for cancer facilities to recruit patients to share their treatment stories and/or concerns. Future studies could improve the reach of recruitment by engaging health care providers or multi-media approaches, and consider scaling the conduct of interviews to a larger geographical region.

Second, the recruitment of participants was intended to include patients from both cancer hospitals and complementary care centres, though only one participant was recruited from a cancer hospital. The other respondents (*n* = 9) were recruited from the same integrated health clinic. This self-selection bias may have encouraged harmonious, positive views of yoga and signalled premature theoretical data saturation because of converging themes and patterns. The experiences or opinions of patients from other cancer care hospitals, including publicly-funded institutions may differ from this sample and should be included in future research.

Third, while the inclusion of men and other tumour types were better represented in this study than existing literature, this sample included experiences from several breast cancer patients (*n* = 5). Furthermore, patients with serious physical limitations or side effects from a recent surgery did not volunteer for this study, so their attitudes or experiences of yoga are not represented here. Also a consequence of self-selection, this sample included only middle age and older adults.

### Implications for yoga research in adult cancer

#### Population

Previous findings suggest yoga use in patient populations is highly correlated with socioeconomic and demographic factors, as well as mental health status and health locus of control and not with patients’ diagnosis [27]. Popular surveys and systematic reviews also indicate adult women, especially breast cancer survivors with higher levels of education and socioeconomic status, participate more frequently in yoga [28, 12]. The results of this study also suggest yoga adherence may be stronger for intrinsically motivated patients and perhaps those already engaged in complementary therapy. Furthermore, the majority of women interviewees in this study had breast cancer (*n* = 5) and completed tertiary education (*n* = 7). If yoga is to be explored as a treatment option for all patients with cancer, then it is also necessary to include participants from socially diverse backgrounds and include a range of socio-demographic groups in future research studies.

Still, this study adds that men could enjoy health and social benefits of practising yoga during cancer treatment. Two men were especially committed to yoga for its non-medical approach to reducing anxiety, improving physical fitness and improving their experience of having cancer. These findings should be explored further with additional research.

#### Characteristics of yoga intervention

Clinical trials of yoga intervention often include expert instruction in a clinical setting and provision of material support for a home-led practice [29–32]. Interviewees reported a yoga-specific environment was important for their comfort and benefits of practice. The degree to which a preferred yoga environment can be transferred to the clinical setting remains largely unknown at this time. Components of yoga intervention that are seemingly important include: 60-minute intervention of breathing techniques, with a variety of physical postures led by expert instructor in a private room, a calming atmosphere with some natural light or window into nature ideal, and a space without any chairs or tables or electrical equipment, with limited background noise and/or smells that could offend patients with sensitivity. The size of yoga’s effect in clinical trials may be underestimated if yoga has been delivered in suboptimal conditions and these findings suggest the contextual meaning of what constitutes the ideal ‘yoga ambience’ is an area for further study.

In other research, online yoga is currently being tested [33] for use in adult cancer. This is a sensible approach to current barriers including accessibility, scheduling conflicts and scalability of yoga interventions in health care. However, findings from this study indicate patients avoid online yoga participation, reportedly due to the loss of a yoga-inspired ambience and quality of in-person instruction as noted in several interviews. Furthermore, this and earlier qualitative analysis [34] suggest yoga in-person increases social interaction and personal motivation to attend, a component possibly compromised during online or home-based practice. The appropriateness of online or video-led yoga is an outstanding question that could be investigated further.

Community-based or public yoga classes were not attended by these cancer patients for fear of stigma and lack of perceived instructor’s awareness of their special needs. The cost of regular yoga classes were prohibitive and a noted limitation for cancer patients. In addition, yoga participants had mixed views as to whether they would do yoga before or after a clinical appointment, stating that timing and illness after conventional treatment sessions were a worry for them.

## Conclusion

As a burgeoning therapeutic intervention in adult cancer, yoga practice could improve aspects of patient psychological, physical and social wellbeing. Patients in this qualitative analysis often experienced yoga as a beneficial complement and antidote to the negative effects of cancer and its treatment. Men and women said they were motivated to do yoga for its healing effects, in body and mind, particularly for strengthening body awareness and stress relief.

This sample of patients frequently attended an instructor-led, restorative yoga class that included training in breathing techniques, physical postures and meditation in a cancer-specific group setting. In addition, patients seeking complementary care in this sample acknowledged the non-medical environment and promotion of self-healing as critical in their healing process. Patients reported affinity for yoga as a complementary therapy, but felt one or more concerns of transportation, scheduling, lack of time and cost were significant barriers to yoga adherence.

Further studies could assist to optimize yoga intervention in adult cancer by comparing the emergent themes in this study, with outcomes of other yoga practices in a larger sample. Future samples in yoga research should endeavor to include younger adults from more diverse socioeconomic and demographic backgrounds of patients undergoing conventional treatment for tumour types other than breast cancer.
